# The *centella asiatica* juice effects on DNA damage, apoptosis and gene expression in hepatocellular carcinoma (HCC)

**DOI:** 10.1186/1472-6882-14-32

**Published:** 2014-01-20

**Authors:** Faridah Hussin, Sima Ataollahi Eshkoor, Asmah Rahmat, Fauziah Othman, Abdah Akim

**Affiliations:** 1 Faculty of Medicine and Health Sciences, Universiti Putra Malaysia, 43400 Serdang, Selangor, Malaysia; 2 Institute of Gerontology, Universiti Putra Malaysia, 43400 Serdang, Selangor, Malaysia

**Keywords:** *Centella asiatica* juice, DNA damage, Apoptosis, Gene expression, Hepatocellular carcinoma (HCC)

## Abstract

**Background:**

This paper is to investigate the effects of *Centella asiatica* on HepG2 (human hepatocellular liver carcinoma cell line). *Centella asiatica* is native to the Southeast Asia that is used as a traditional medicine. This study aims to determine the chemopreventive effects of the *Centella asiatica* juice on human HepG2 cell line.

**Methods:**

Different methods including flow cytometry, comet assay and reverse transcription-polymerase chain reaction (RT-PCR) were used to show the effects of juice exposure on the level of DNA damage and the reduction of cancerous cells. MTT assay is a colorimetric method applied to measure the toxic effects of juice on cells.

**Results:**

The *Centella asiatica* juice was not toxic to normal cells. It showed cytotoxic effects on tumor cells in a dose dependent manner. Apoptosis in cells was started after being exposed for 72 hr of dose dependent. It was found that the higher percentage of apoptotic cell death and DNA damage was at the concentration above 0.1%. In addition, the juice exposure caused the reduction of c-myc gene expression and the enhancement of c-fos and c-erbB2 gene expressions in tumor cells.

**Conclusions:**

It was concluded that the *Centella asiatica* juice reduced liver tumor cells. Thus, it has the potential to be used as a chemopreventive agent to prevent and treat liver cancer.

## Background

Hepatocellular carcinoma (HCC) is one of the most common causes of cancer and cancer-related deaths worldwide that has been increased in the recent years [1]. HCC usually occurs due to viral hepatitis, hemochromatosis, alcohol-related liver disease, autoimmune hepatitis, nonalcoholic steatohepatitis (NASH), primary biliary cirrhosis (PBC) and primary sclerosing cholangitis (PSC) [2]. HCC cancer mostly affects Southeast Asia and sub-Saharan Africa [1]. Scientists have demonstrated that more than two-thirds of cancer cases can be avoided or prevented by lifestyle modifications [3]. Herbal medicine can be used as a part of lifestyle to prevent and treat diseases [4], therefore, further studies are needed to clarify new biologically active compounds in the medicinal plants [5]. *Centella asiatica* or pegaga is one of the local medicinal plants in swampy areas including India, Sri Lanka, South Africa, China, the western South Sea Island, Australia, Madagascar, southern United States, the Southeast Asia such as Malaysia and Indonesia as well as insular and continental tropical America [6]. This plant has been claimed to have various medicinal effects that is consumed in different forms including tea, juice, pills and capsules [7]. It has been used to treat several problems such as insanity, asthma, leprosy, ulcers, eczema and wound healing. *Centella asiatica* has triterpene glycosides such as centellasaponin, asiaticoside, madecassoside, sceffoleoside, asiatic acid and madecassic acid. The most abundant triterpene glycoside in the extracted juice is asiaticoside, which can be transformed into asiatic acid. This compound has cytotoxic effects on fibroblast cells as well as the ability to induce apoptosis in different sorts of cancer [8].

Biological activity of compounds present in the *Centella asiatica* plant can alter the expression level of different genes [9]. For example, the extracted juice can change the expression of c-myc, c-fos and c-erbB2 oncogenes in exposed cells. C-myc gene promotes cell cycle progression in cells [10,11]. C-fos gene is normally involved in cell cycle regulation, cell differentiation and transformation [12] that is induced by c-erbB2 gene [13]. This study was conducted to identify the cytotoxic and chemopreventive effects of *Centella asiatica* juice on HCC cells assessed at different concentrations.

## Methods

### Chemicals

Two cell lines, human normal liver cell line (Chang liver) and hepatocellular carcinoma cell line (HepG2) were purchased from American Type Culture Collection (ATCC), Rockville, Maryland, USA. Furthermore, the materials of Dulbecco’s modified Eagle’s medium (DMEM), dimethyl sulfoxide (DMSO), fetal calf serum, penicillin, streptomycin, PBS-EDTA, trypsin and propidium iodide were bought from Sigma Chemical Co. (Gibco, USA). Trypan blue dye and 3-(4,5-dimethylthiazol-2-yl)-2,5-diphenyltetrazolium bromide (MTT) were purchased from Sigma Chemical Company (St. Louis, MO, USA). In addition, the falcon flasks for cell culture were taken from Nunc Co. Denmark. The MasterPure^TM^ RNA Purification Kit was purchased from Epicentre Technologies (Becton Dickinson Corp., Rutherford, New Jersey, USA) to purify RNA. The used chemicals were with the highest pure grade available.

### Preparation of centella asiatica juice

The project was undertaken at the Faculty of Medicine and Health Sciences in Universiti Putra Malaysia (UPM). Optimum formulation and processing parameter for herbal juice preparation was selected from the response surface methodology analysis. *Centella asiatica* plant (Voucher No. SK 533/03) locally named pegaga cina, a native and well known plant was collected from an Herbal Farm in Melaka, Malaysia. After collection, plant was weighed, washed, cut into small pieces and then deposited at the Institute of Bioscience, UPM. It was mixed with filtered water containing 0.1% (w/w) sodium metabisulphite. After that, the dried plant was grounded into very fine particles using mechanical grinder. 14% of puree was mixed with 14% honey, 0.2% (w/w) xanthan gum and homogenized using Homogenizer IKA II for 5 min. Mixture was pasteurized at 80°C for 3 min using jacketed heater and final products were hot-filled into sterilized glass bottle, cooled to a room temperature under running water and kept chilled for analysis. The herbal extract sterilization can be done by using both high temperature and pressure.

### Cell culture and treatment

Cell cultures were carried out in the Microbiological Safety cabinet ERLA Esc Series Class II. Cells grown in DMEM supplemented with 10% fetal bovine serum (GIBCO BRL), 1% penicillin and streptomycin were incubated in a humidified incubator with 5% CO2 and 95% air at 37°C. Media of the culture environment was changed frequently each three days. The cell cultures were regularly examined using inverted microscope. For treatment, the suitable amount of cells (about 1×10^6^ cells/ml) was pipetted into the cell culture flasks (Nunc, Denmark), which were provided three for each concentration. Then, these flasks were incubated in a 5% CO_2_ incubator (Sanyo, Japan) at 37°C for 24 hr. HepG2 cells were treated with the *Centella asiatica* juice at 0.01, 0.1, and 1% concentrations. Cells were left in a 5% CO2 incubator at 37°C for 72 hr. The harvested cells in the flasks were used to conduct experiments.

### MTT assay

The MTT assay was used to analyze cell proliferation among cells [14]. HepG2 and Chang cell lines harvested in flasks were detached by trypsin and stained with trypan blue to determine the viability by counting the cells using a haemocytometer under the inverted microscope (Olympus CK40, Japan). Cells were diluted with DMEM to yield a suspension at a concentration of 1×10^5^ cells/ml and then 100 μl of suspension was pipetted into a 96-well micro titer plate (Nunc, Denmark). After that, this plate was incubated in a 5% CO2 incubator at 37°C for 24 hr to allow the cells attached to well. After incubation, cells were treated with 100 μl of sterile sample (filter using 0.2 μm; Schleicher and Schuell) prepared at different concentration levels of 0.001, 0.01, 0.1, 1.0 and 10%. The 96 well-plate was then kept in a CO_2_ incubator for 24, 48 and 72 hr. After that, 10 μl of MTT was pipetted into wells and incubated again for 4 hr. Finally, 100 μl of Dimethyl sulfoxide (DMSO) was added into wells and the absorbance was read using Microplate absorbance reader Anthos Zenyth 340 s at wavelengths between 500 and 600 nm. The calculation of absorbance for each concentration of juice was compared with untreated control.

### Flow cytometry

After the treatment of cells, DNA content was measured using Flow cytometer Becton Dickinson FACStarPLUS. Cells were initially treated by different concentrations of the *Centella asiatica* juice including 0.01, 0.1, and 1% and then incubated in 5% CO2 at 37°C for 72 hr. Cells were fixed by adding the absolute ethanol, followed by adding 200 μg/ml of DNase-free Rnase A and incubated at 37°C for 30 min. After this step, cells were permeabilized using Trition X-100, combined with 100 μl of 1 mg/ml propidium iodide as a light sensitive and incubated at room temperature for 5-l0 min. Samples (untreated and treated cells) were placed into 12 × 75 mm Falcon tubes and read using a Becton Dickinson FACStarPLUS (Becton Dickinson, Franklin Lakes, NJ). The presence of cells in sub-G1 phase was indicator of apoptotic cell death.

### Comet assay

Comet assay was used to evaluate DNA damage level in the treated cells compared to controls. This experiment was performed according to a modified protocol, which was basically suggested by McKelvey-Martin and co-workers [15]. The assay was carried out under dimmed light to prevent cell damage from UV [16]. Frosted microscope slides were covered with 80 μl of 1% normal melting agarose (NMA) in Tris-acetate-EDTA (TAE) buffer at 45°C, then covered with cover slip and kept at 4°C for 5 min until agarose had been solidified. Approximately 75 μl of 0.5% low melting point agarose (LMA) at 37°C was added to the pellet of cells suspended in 10 μl of PBS and then the cell suspension was rapidly pipetted onto the first (NMA) agarose layer of slides. These slides were immersed in freshly prepared, cold lysis solution added with 1% triton X-100 at 4°C for 1 hr in a dark place. After that, slides were removed, drained and immersed in electrophoresis buffer (10 N NaOH, 200 mM Na_2_EDTA, pH > 12) at 4°C for 20-60 min in the dark to unwind DNA. Then, slides were placed flat on a gel tray with aligned equidistant from the electrodes. The electrophoresis was conducted at 25 V adjusted to 300 mA for 20 min. After that, slides were stained with 50 μl of 20 μg/ml ethidium bromide and then viewed using fluorescence microscope.

Analysis was performed immediately after staining under a Leica Epifluorescence microscope equipped with an excitation filter of 515-560 nm (Bellows Falls, VT, USA) with magnification of 400×. For each sample, 15-50 of cells were analysed. Slides were scored by moving from top to bottom slide along to avoid analysis of same comet twice. Parameters of tail length (distance from the head center to the end of the tail) and tail migration (distance from the end of the head to the end of the tail) were used to estimate DNA migration.

### Gene expression and RT-PCR

The amplification and detection of mRNA expression of oncogenes in HepG2 cell line was done using primer of oncogenes and primer of housekeeping genes. Total RNA was extracted from the cells treated with the *Centella asiatica* juice. MasterPure^TM^ RNA Purification Kit from Epicentre Technologies (Becton Dickinson Corp., Rutherford, New Jersey, USA) was used to purify RNA. The extracted RNA was treated with DNase and then used to synthesize cDNA by One Step RT-PCR according to RT-PCR Premix protocol (Mbiotech, Korea). In this study, c-myc, c-fos and c-erbB2 oncogenes were studied. Housekeeping genes including ß-actin and 15 s were selected to be internal positive controls.

PCR primers for c-myc gene were obtained from a published article [17]. The primers used for c-fos, c-erbB2, 15 s and ß-actin genes were designed. The sense primers for c-fos, c-erbB2, c-myc, 15 s and ß-actin genes were 5′-GGATAAGATGGCTGCAGCCAAGTGC-3′, 5′-GATGTATTT-GATGGTGACCT-3′, 5′-CAAGAGGCGAACACACCACGTCT-3′, 5′-TTCCGCAAGTTCAC-CTACC-3′ and 5′-CGTGGGCGCCCTAGGCACCA-3′ respectively. The antisense primers for c-fos, c-erbB2, c-myc, 15 s and ß-actin genes were 5′-AAGGAAGACGTGTAAGCAGTGCAG-C-3′, 5′-ATCTGGCTGGTTCACATATT-3′, 5′-AACTGTTCTCGTCGTTTCCGCAA-3′, 5′–C-GGGCCGGCCATGCTTTACG-3′ and 5′-TTGGCCTTAGGGTTCAGGGGGG-3′ respectively.

The expression of oncogenes was assessed at the low, medium and high concentrations of juice. Oncogenes including c-myc, c-fos and c-erbB2 were amplified along with either 15 s or β-actin housekeeping genes to detect their expression levels. Each PCR reaction included 1 ng-2 μg total RNA, 1 μl of each primer (10pmol/μl), 10 μl of One Step RT-PCR Premix Master Mix (5×) and 7 μl of RNase-free water in a single PCR reaction tube to produce a volume of 20 μl. The reverse transcription was performed at 42°C within 30 min in one cycle followed by RT inactivation and pre-denaturation at 96°C for 3 min. The PCR reaction was continued under the same conditions within 30 cycles for all samples. The cycles were settled on a denaturation stage at 94°C for 30 s, annealing temperature at 50-60°C for 30 s found through the gradient PCR, an extension phase at 72°C for 60 s and a final extension at 72°C for 10 min followed the reaction last cycle.

After PCR was finished, samples were stored at 4°C until use. A negative control without DNA template was carried out in each run. After the completion of PCR, products were analyzed by running on 1.5% agarose gel electrophoresis. In addition, DNA ladder of 100 bp (Bioline, USA) was used to identify the sizes of products. The visualization of DNA was done by placing the gel onto UV light source. The quantity of RT-PCR bands were analyzed based on the intensity under Gel Doc 2000 software (BIO-RAD). The ratios of target genes to reference genes (housekeeping gene) were calculated and compared.

### Statistical analyses

Data were analysed using the Statistical Package for the Social Sciences (SPSS) software version 19.0 (Chicago, IL, USA). The critical level for rejection of the null hypothesis was considered to be a p value of 5%, two-tailed. Values were expressed as mean ± SD. The Data were statistically analyzed using one way-ANOVA followed by Tukey’s post Hoc t-test analysis.

## Results

**Figure 1:**
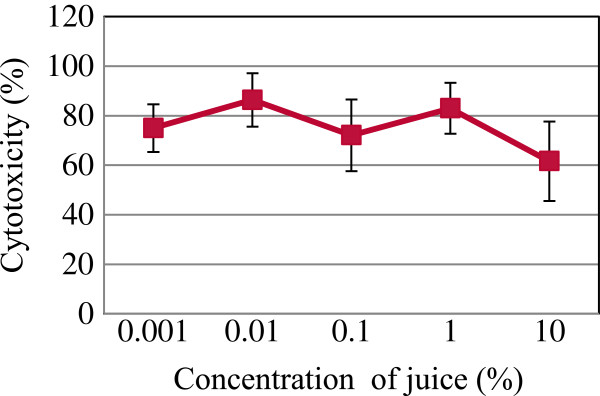
**The cytotoxic effects of *Centella asiatica *juice on Chang liver cell line via MTT-assay.** Cells were seeded in 96-well plates at a density of 10^5^ and treated with the filtered *Centella asiatica* juice (0.001-10%) for 72 hr. Values are presented as means (n = 3) ± S.E.

**Figure 2:**
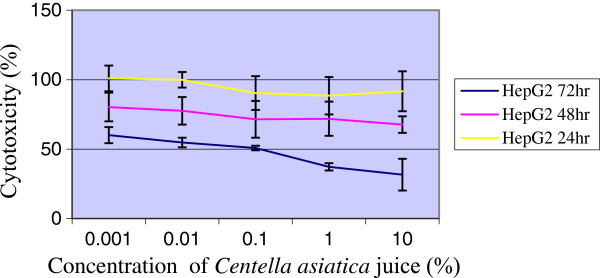
**The cytotoxic effects of *Centella asiatica *juice on HepG2 cell line via MTT-assay.** Cells were seeded in 96-well plates at a density of 10^5^ and treated with filtered *Centella asiatica* juice (0.001-10%) for 24, 48 and 72 hr. Values are presented as means (n = 3) ± S.E.

It was found that the increase in the doses of *Centella asiatica* juice up to 10% had no cytotoxic effects against Chang cell line when cells were incubated up to 72 hr (Figure 1). The results showed that the inhibitory effects of juice on proliferation of HepG2 cancer cell line was associated with concentration doses and exposure duration time. The cytotoxicity started at a concentration dose of 0.1% when HepG2 cells were treated and incubated up to 72 hr (Figure 2). This effect showed a chemopreventive activity of juice against liver tumor cells. Furthermore, flow cytometry showed the rate of cell death and growth arrest in the treated cells with juice.

**Figure 3:**
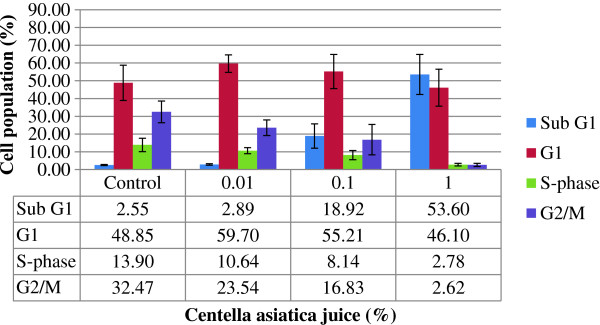
**Cell cycles profiles of HepG2 cells untreated and treated with the *Centella asiatica *juice (CAJ) and subsequently stained with propidium iodide.** Cells were exposed to 0.01, 0.1 and 1.0% of CAJ for 72 hr.

**Figure 4:**
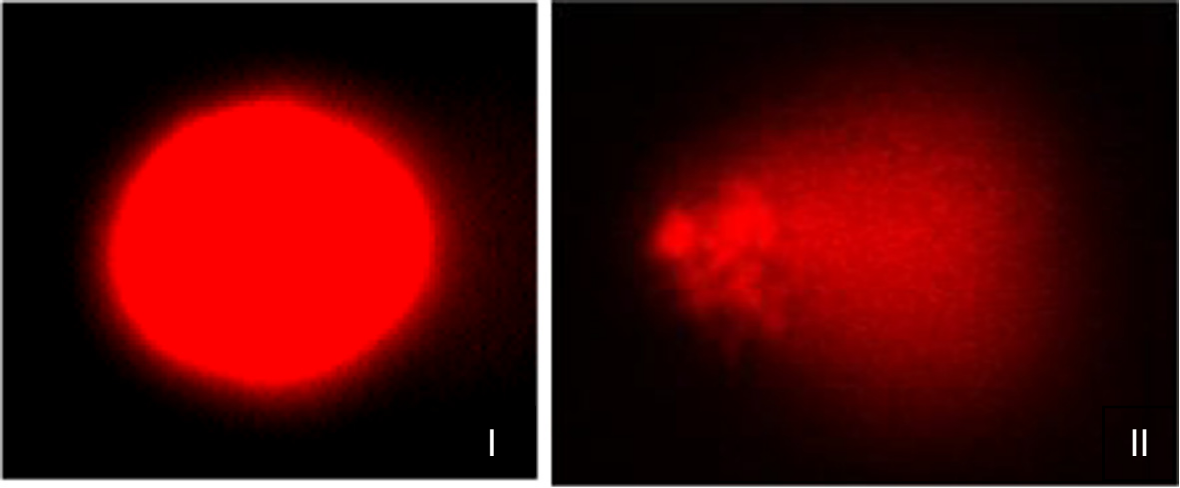
Fluorescence image of red-stained comets from control (I) and juice treated (II) HepG2 cells (20× magnification).

Apoptosis increased in HepG2 cells treated with juice when they were incubated for 72 hr. The cell death in HepG2 cell line was 8 and 21 times more than controls when they were treated with respective concentrations 0.1 and 1%. By increasing the juice concentrations, cell population in the G2/M arrest and S-phase decreased. In addition, the juice treatment increased cells in the Sub G1 phase between 3% and 54% and decreased cells in the G2/M phase between 33% and 3%. Furthermore, the G1 phase slightly increased after the treatment with 0.01% concentration and gradually decreased with the increase of concentrations (Figure 3). The treatment with 0.1 and 1% concentrations of juice significantly increased DNA damage in HepG2 cells (p < 0.05) (Figure 4).

**Figure 5:**
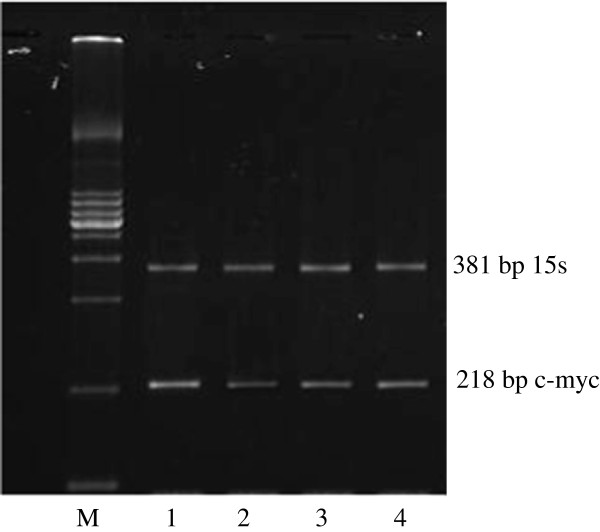
**Expression of c-myc oncogene in untreated (1) and treated (2, 3, 4) HepG2 cancer cell lines with 0.01, 0.1, 1.0% *Centella asiatica *juice respectively for 72 hr. Note the expression of c-myc band before (1) and after treatment (2, 3, 4).** Lane M is the DNA ladder for marker and act as comparison to the oncogene band.

**Figure 6:**
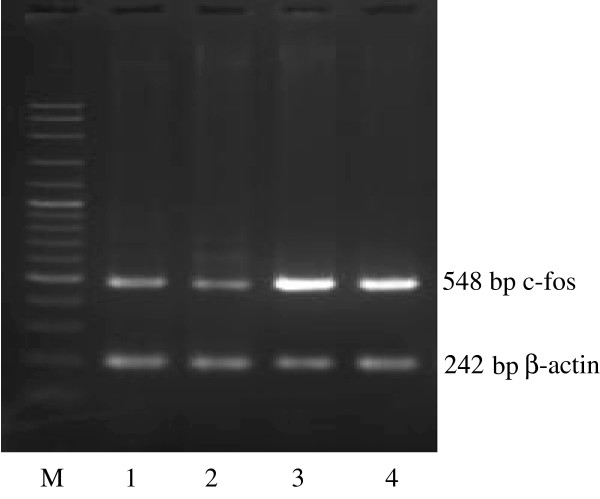
**Expression of c-fos oncogene in untreated (1) and treated (2, 3, 4) HepG2 cancer cell lines with 0.01, 0.1, 1.0% *Centella asiatica *juice respectively for 72 hr. Note the expression of c-fos band before (1) and after treatment (2, 3, 4).** Lane M is DNA ladder for marker and act as comparison to the oncogene band.

**Figure 7:**
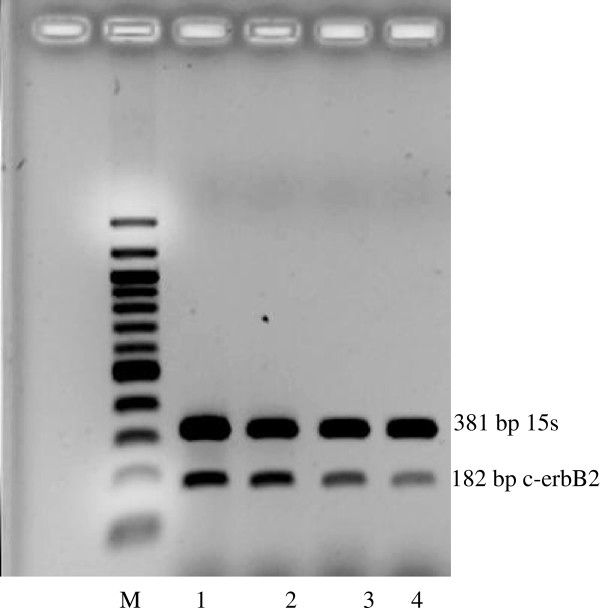
**Expression of c-erbB2 oncogene in untreated (1) and treated (2, 3, 4) HepG2 cancer cell lines with 0.01, 0.1, 1.0% *Centella asiatica *juice respectively for 72 hr. Note the expression of c-erbB2 band before (1) and after the treatment (2, 3, 4).** Lane M is DNA ladder for marker and act as comparison to the oncogene band.

C-myc, c-fos and c-erbB2 oncogenes were detected at 218, 584 and 182 bp respectively whereas the 15S and β-actin housekeeping genes were detected at 381 and 242 bp respectively (Figures 5, 6 and 7). After the treatment with 0.01, 0.1 and 1.0% concentrations of the *Centella asiatica* juice, c-myc gene was suppressed in HepG2 cells. The expression of c-fos and c-erbB2 genes increased at the concentrations of 0.1% and above (Table 1). The treatment of cells with juice showed that the expression level of c-fos increased by 83% in both concentrations of 0.1 and 1.0%. Meanwhile, these respective concentrations increased the expression of c-erbB2 gene by 22% and 16%.

**Table 1:** Ratio of target genes to reference genes expression in untreated and treated

**Concentration of *Centella asiatica *** **juice (%)**	**Expression of oncogenes in HepG2 (INT/mm**^**2**^**)**		
	C-myc	C-fos	C-erbB2
0	1.43	1.2	2.54
0.01	0.69	1.0	2.16
0.1	0.69	2.2	3.11
1.0	0.94	2.2	2.95

HepG2 cell line with *Centella asiatica* juice.

## Discussion

Plant products contain natural anticancer and chemopreventive agents, which can protect body against cancer [18]. The cytotoxic effects of *Centella asiatica* juice on HepG2 cell line suggests the susceptibility of cells for being treated with juice. Such effect of juice is probably due to the compounds having cytotoxic and anti-tumour properties [19]. The *Centella asiatica* compounds can cause apoptosis [20,21] to protect cells against neurotoxicity. Furthermore, apoptosis can inhibit the promotion [22] and invasion [23] of cells in different types of tumors [24,25]. Such effects were related to dose concentration and exposure duration time. The protective effects of juice [26] against cancer is most likely due to the effects of antioxidants [27,28] and apoptotic cell death [20,21]. Thus, *Centella asiatica* can be used as a potential natural chemopreventive agent to combat liver cancer. Our results confirmed the previous research [29] indicating that the *Centella asiatica* juice has no potent cytotoxic ability and toxic effects on healthy cells.

The effects of juice were assessed on c-myc, c-fos and c-erbB2 genes as genes involved in cell cycle control. It was found that the *Centella asiatica* juice reduced c-myc gene in HepG2 cells. It seems that this condition was a response to sustain DNA damage in HepG2 cells during DNA fragmentation in apoptosis [30,31]. Down regulation of c-myc gene may affect apoptosis due to the following conditions; serum starvation, chemically induced and exposed to viruses [32,33]. On the other hand, up-regulation of c-myc oncogene has been found in human hepatoma cells as well [34]. Such variety suggests the contribution of different expression of c-myc gene to the regulation of apoptotic cell death [35], which can be related to the type of stimuli required for starting and efficient induction of apoptosis [36,37].

In the current study, the treatment of HepG2 cells with the *Centella asiatica* juice increased c-fos gene expression, which is more likely due to stimulating effects of juice [38-40] on the increased level of apoptosis, DNA breakage [38-40], the apoptotic late response of cells [41] and c-myc-mediated apoptosis [34]. In addition, the *Centella asiatica* juice increased the expression of c-erbB2 gene in HepG2 cells probably due to the association of c-erbB2 gene with differentiation and apoptosis process. It has been documented that a complex integration of signal transduction pathways of proto-oncogenic proteins can mediate apoptosis process in different cells through decreased or increased expression, which depends on cell-type specificity and nature of apoptotic stimuli such as drug or compounds [42]. The induction of apoptosis in HepG2 can be explained by the interference of *Centella asiatica* juice with oncogenes in liver cancer cells. It shows that genetic alterations and the impairment of oncogene activities may associate with apoptosis [42].

## Conclusions

It was concluded that the *Centella asiatica* juice inhibited the proliferation of a malignant HepG2 cell line through apoptosis or programmed cell death. Flow cytometry and comet assay analysis showed that juice increased DNA damage in HepG2 cells when they were exposed for 72 hr. In addition, juice reduced the level of c-myc gene expression but increased the level of c-fos and c-erbB2 genes in HepG2 cells. Such changes increased apoptosis in the liver tumor cells. It was concluded that the *Centella asiatica* juice can maintain the health of liver and reduce the incidence of liver cancer. Further investigations are needed in future to identify the exact effects of this juice on HepG2 cells regarding the reduction and treatment of liver cancer.

## Abbreviations

ATCC: American type culture collection; DMEM: Dulbecco’s modified eagle’s medium; DMSO: Dimethyl sulfoxide; HCC: Hepatocellular carcinoma; LMA: Low melting point agarose; NASH: Nonalcoholic steatohepatitis; NMA: Normal melting agarose; PBC: Primary biliary cirrhosis; PSC: Primary sclerosing cholangitis.

## Competing interests

Authors declare that there are no competing interests in relation to this manuscript.

## Authors’ contributions

FH: preparation of draft, contribution to conception, design, acquisition of data, analysis and interpretation of data. SAE: involved in conception, drafting the manuscript and revising it critically. AR: contribution to conception, design, data analysis and final approval of the version for publishing. FO: contribution to conception and design of study. AA: contribution to conception and design of study. All authors read and approved the manuscript.

## Pre-publication history

The pre-publication history for this paper can be accessed here:

http://www.biomedcentral.com/1472-6882/14/32/prepub
